# Prevalence and Intensity of Periodontal Disease in Individuals with Metabolic Syndrome

**DOI:** 10.25122/jml-2020-0073

**Published:** 2020

**Authors:** Tatiana Anatolyevna Hlushchenko, Victor Markianovich Batig, Anatoly Vasylovych Borysenko, Olha Mykhaylivna Tokar, Iryna Viktorivna Batih, Olena Mykolayivna Vynogradova, Oksana Grygorivna Boychuk-Tovsta

**Affiliations:** 1. Department of Therapeutic Stomatology, Bukovinian State Medical University, Chernivtsi, Ukraine; 2. Department of Therapeutic Stomatology, O.O. Bohomolets National Medical University, Kyiv, Ukraine; 3. Department of Pediatric Dentistry, Bukovinian State Medical University, Chernivtsi, Ukraine; 4. Department of Therapeutic Dentistry, Faculty of Postgraduate Education, Danylo Halytsky Lviv National Medical University,Lviv, Ukraine; 5. Ivano-Frankivsk National Medical University, Ivano-Frankivsk, Ukraine

**Keywords:** Metabolic syndrome, periodontal disease, pathogenetic therapy

## Abstract

Metabolic syndrome is one of the actual problems of modern medicine because of its high prevalence in the general population and its essential role in the development and progression of cardiovascular diseases.

In the last decade, studying the relationship between metabolic syndrome and periodontal diseases has attracted many scientists’ attention. Based on this, the study of the clinical features of periodontal diseases in the early stages of metabolic syndrome is relevant and necessary for timely and successful pathogenetic therapy.

The purpose of our study was to investigate and analyze the prevalence and intensity of periodontal disease in people with metabolic syndrome. To solve this goal, we surveyed 190 people with metabolic syndrome who were registered at the endocrinological clinic in Chernivtsi. They formed the main observation group. The comparison observation group included 90 people without metabolic disorders. The age of the patients ranged from 25 to 55 years. Periodontal disease was detected in 155 of 190 patients with metabolic syndrome (81.58 ± 2.82%). In 90 patients without endocrinological pathology, the prevalence of periodontal disease was 1.2 times lower (65.56 ± 5.04%; p <0.01). Generalized periodontitis prevailed in the structure of periodontal diseases in patients with metabolic syndrome: 26.45±3.56% cases were in the second stage of generalized periodontitis (GP), and 21.94±3.33% in the third stage of GP, р<0.01. Therefore, the metabolic syndrome, as a state with a high risk of diabetes development, creates conditions for the formation and rapid progression of inflammatory-destructive periodontal lesions.

## Introduction

Metabolic syndrome is one of the actual problems of modern medicine because of its high prevalence in the general population and its important role in the development and progression of cardiovascular diseases [1-4]. The metabolic syndrome includes abdominal obesity, dyslipidemia, arterial hypertension, and disorders of carbohydrate metabolism, and its pathogenetic essence is the phenomenon of insulin resistance [5-9]. In the last decade, studying the relationship between metabolic syndrome and periodontal diseases has drawn the attention of many scientists [10-15]. Disorders that are components of the metabolic syndrome underlie the mechanism of development of many pathological processes, such as hypertension, coronary heart disease, obesity, gout, and others [16-20]. Organs and structures of the oral cavity, including the periodontium, are also involved in the pathological process [21-25]. At the same time, inflammatory-dystrophic changes in the periodontium are directly dependent on such factors as age, disease severity, conducted therapy [26-29]. On this basis, it is relevant and necessary to study the features of the clinical manifestation of periodontal diseases in the early stages of metabolic syndrome for timely and successful pathogenetic therapy.

The purpose of our study was investigation and analysis of prevalence and intensity of periodontal disease in people with metabolic syndrome.

## Material and Methods

To solve this goal, we surveyed 190 people with metabolic syndrome who were registered at the endocrinological clinic in Chernivtsi, and they formed the main group. The comparison group included 90 people without metabolic disorders. The age of surveyed people ranged from 25 to 55 years. To determine metabolic syndrome, endocrinologists used the criteria proposed by the World Health Organization (WHO) in 1998. According to this criteria, metabolic syndrome includes a violation of tolerance to glucose or type 2 diabetes and/or insulin resistance, combined with two or more of the following criteria: increase in blood pressure to 160/90 mmHg; increased plasma triglycerides levels (greater than 1.7 mmol/l) and/or low levels of high-density lipoprotein cholesterol (less than 0.9 mmol/l in men and less than 1.0 mmol/l in women) [9].

The diagnosis of periodontal diseases was classified by MF Danilevsky in 1994.

## Results

The results of the prevalence of periodontal diseases in people with metabolic syndrome are presented in Table. 1. According to the data, periodontal disease was detected in 155 of 190 patients with metabolic syndrome, which was 81.58 ± 2.82%. In 90 patients without endocrinological pathology, the prevalence of periodontal disease was 1.2 times lower (65.56 ± 5.04%; p <0.01).

**Table 1:** Prevalence of periodontal disease in the observation groups.

Periodontal condition	Main group n = 190		Comparison group n = 90	
	Abs. number	%	Abs. number	%
**Intact periodontal tissue**	35	18.42 ± 2.82	31	34.44 ± 5.04*
**Periodontal disease**	155	81.58 ± 2.82	59	65.56 ± 5.04*

Note: * the significance of the difference between the main group and the comparison groups (p < 0.01).

**Figure 1:**
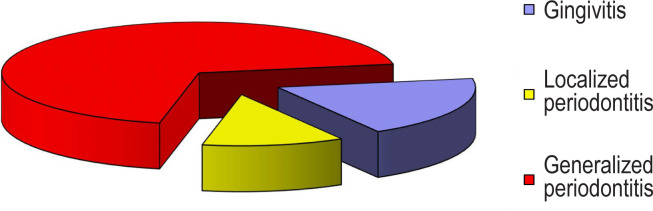
Nosological forms of periodontal diseases in the main group.

Generalized periodontitis prevailed in the structure of periodontal diseases in patients with metabolic syndrome (Figure 1).

The highest percentage was found in the advanced stages of the disease: 26.45±3.56% cases were in the second stage of generalized periodontitis (GP), and 21.94±3.33% in the third stage of GP, р<0.01.

**Figure 2:**
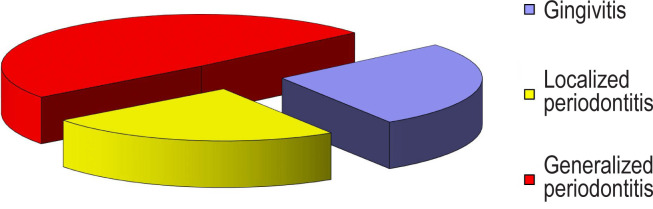
Nosological forms of periodontal diseases in the comparison group.

In the group of people without metabolic disorders, the situation was the opposite: the initial stages of periodontal disease were diagnosed in the largest percentage of the patients (Figure 2).

Thus, gingivitis was detected in 27.12 ± 5.84% of patients in the comparison group, which was 1.4 times more than in the main group, p <0.01 (Table 2). Localized periodontitis was found in 23.73 ± 5.59% of the patients in the comparison group, p <0.05. The number of cases in the initial stage of generalized periodontitis in the comparison group was 1.06 times higher than in the main group (р<0.01). However, the number of cases in the second stage of generalized periodontitis in patients without metabolic disorders was 18.65 ± 5.11% and was 1.4 times lower than in patients with metabolic syndrome (p <0.01). The lowest percentage in the structure of periodontal diseases in the comparison group (8.47 ± 3.66%) were in the third stage of generalized periodontitis. In contrast, in the main group, the percentage of people in the third stage of GP was 2.6 times higher (21.94 ± 3.33 %, p <0.01).

**Table 2:** Structure of periodontal diseases in the observation groups.

Periodontal condition	Main group n = 190		Comparison group n = 90	
	Abs. number	%	Abs. number	%
**Gingivitis**	30	19.35 ± 3.18	16	27.12 ± 5.84**
**Localized periodontitis**	18	11.61 ± 2.58	14	23.73 ± 5.59*
**GP initial stage**	32	20.65 ± 3.26	13	22.03 ± 5.44**
**GP stage I**	41	26.45 ± 3.56	11	18.65 ± 5.11**
**GP stage II**	34	21.94 ± 3.33	5	8.47 ± 3.66**
**GP stage III**	155	100	59	100

Note: * the significance of the difference between the main group and the comparison group (p<0.01).

One of the objectives of our study was the investigation of the prevalence and intensity of periodontal tissue diseases in patients with metabolic syndrome regarding the age aspect (Table 3). In the 25-34 age range, periodontal diseases were found in 64.15 ± 5.63% of patients with metabolic syndrome, which was 1.3 times more than those without metabolic disorders (47.62 ± 11.12 %, p <0.01). In the age range of 35-44 years, the number of persons with periodontal diseases in the main group increased to 83.08 ± 3.12%.

**Table 3:** Prevalence of periodontal disease in the observation groups depending on age.

**Age groups (years)**	**Main group**			**Comparison group**		
	Number of surveyed	With periodontal diseases	%	Number of surveyed	With periodontal diseases	%
25 – 34	53	34	64.15 ± 5.63	21	10	47.62 ± 11.12*
35 – 44	65	54	83.08 ± 3.12	33	21	63.64 ± 7.10*
45 – 55	72	67	93.05 ± 3.12	36	28	77.78 ± 6.40*
Total	190	155	81.58 ± 4.61	90	59	65.56 ± 4.20*

Note: * the significance of the difference between the main group and the comparison groups (p<0.01).

In the comparison group, an increase in the percentage of patients with periodontal pathology was observed. However, the number of patients was 1.3 times lower than in the main group (p<0.01). With increasing of age up to 44-55 years, 93.05 ± 3.12% of cases of periodontal diseases were observed in patients with metabolic syndrome, which was 1.4 times more than in persons without metabolic disorders (77.78 ± 6.40%, p <0.01).

On average, periodontal disease was observed in 81.58 ± 2.82% of patients with periodontal metabolic syndrome, while in patients without metabolic disorders, the percentage of periodontal disease was 1.2 times lower (65.56 ± 4.20%), p <0.01).

Intact periodontal was detected only in 18.42 ± 2.82% of patients with metabolic syndrome.

## Discussions

To study the prevalence and intensity of periodontal disease in people with metabolic syndrome, we surveyed 190 people with metabolic disorders who were registered at the endocrinological clinic in Chernivtsi. These patients formed the main group. The comparison group included 90 people without metabolic disorders. The age of the surveyed patients ranged from 25 to 55 years. According to the obtained data, periodontal disease was detected in 155 of 190 patients with metabolic syndrome, which was 81.58 ± 2.82%. In 90 patients without endocrinological pathology, the prevalence of periodontal disease was 1.2 times lower (65.56 ± 5.04%; p <0.01). Generalized periodontitis prevailed in the structure of periodontal diseases in patients with metabolic syndrome, with the highest percentage being found in advanced stages of the disease: 26.45 ± 3.56% of cases were in the second stage of GP, 21.94 ± 3.33% in the third stage of GP, p <0.01. With increasing age, the percentage of people with periodontal disease with metabolic syndrome increased to 93.05 ± 3.12%.

## Conclusion

Thus, higher prevalence and intensity of periodontal tissue diseases were observed in patients with metabolic syndrome than in patients without metabolic disorders. Regarding the structure of periodontal diseases, severe stages of periodontal diseases prevailed in patients with metabolic syndrome. The progression of periodontal lesions was faster compared to patients without metabolic disorders. Therefore, the presence of metabolic syndrome, as a condition with a high risk of diabetes, creates the conditions for the formation and rapid progression of inflammatory-destructive periodontal lesions.

## Conflict of Interest

The authors declare that there is no conflict of interest.
